# Assessing the Impact of Simplified Language on a Patient-Facing Pharmacogenetic Report: A User Comprehension Study

**DOI:** 10.3390/jpm15060247

**Published:** 2025-06-12

**Authors:** Russell Amato, Nicole M. Del Toro-Pagan, Harris Nguyen, Jordan Plummer, Katie Pizzolato, David Krause, Daniel Dowd

**Affiliations:** Clinical Research & Development and Medical Affairs, Genomind, Inc., King of Prussia, PA 19406, USA; ndeltoro-pagan@genomind.com (N.M.D.T.-P.); jplummer@genomind.com (J.P.); kpizzolato@genomind.com (K.P.); ddowd@genomind.com (D.D.)

**Keywords:** CYP450, patient-centered care, health literacy, pharmacogenetics, medication safety, medication management, precision medicine, pharmacogenomics

## Abstract

**Background:** Pharmacogenetics (PGx) is the science of assessing how genetic variation affects drug efficacy, tolerability, and safety. While PGx is an emerging discipline which is becoming standard of care, many providers have misunderstandings about its utility. This is even more of a problem for patients, who may perceive that there is a single drug that is “right” for them. The primary objective of this study was to evaluate consumer comprehension of a newly developed patient-facing PGx report. **Methods:** In this study, we adapted a commercial pharmacogenetic test (Genomind Professional PGx) into a report intended to be more comprehensible to the consumer. The initial translation of the clinical terminology used in the PGx report, into lay terminology was conducted by PharmDs and PhDs who have collectively provided over 20,000 PGx consults to date. These reports were then evaluated with readability scoring software to ensure each translation’s complexity remained ≤8th-grade reading level. A total of 107 participants were recruited to conduct the initial analysis with a goal of achieving a 90% comprehension rate using the Genomind consumer comprehension survey. These participants were also given a modified Minnesota Assessment of Pharmacogenomic Literacy (MAPL™) both before and after the Genomind comprehension survey to assess overall PGx literacy. **Results:** Ninety-eight (98) out of 107 research participants scored one or zero questions incorrectly, translating to >90% comprehension score on the Genomind consumer comprehension survey. These participants also demonstrated a significant increase in overall pharmacogenetic literacy, as assessed by MAPL after viewing the consumer report and survey. **Conclusions:** This study found that translating pharmacogenetic test results into lay language may provide individuals with a greater understanding of how their DNA may impact prescribed medications.

## 1. Introduction

Advances in health information systems and standards have allowed consumers to access and control their medical data, with the 21st Century CURES Act codifying this [1]. However, the vast amount of data generated by genomics laboratories does not necessarily provide useful understanding for lay individuals, even when such data are available to them. Technical language and jargon may limit the utility of such data [2]. Furthermore, interpreting genetic test results, particularly in the realm of pharmacogenetics, where terminology has only recently begun to be standardized, can be difficult for patients [3,4,5,6,7]. Limited health literacy poses a particular challenge when communicating genetic information, and even individuals with a post-secondary education have numeracy gaps that impact the understanding of genetic risk [8,9]. Conversely, well-designed patient-facing material may help to improve patient understanding, interpretation, and the communication of genetic test results, with various approaches having been described, including interpretive summaries, patient user guides, and completely revised patient-friendly reports [10,11].

Pharmacogenetics (PGx) is the science of assessing how genetic variation affects drug efficacy, tolerability, and safety. PGx is a tool that is increasingly being utilized in mental health, oncology, and cardiovascular disease to facilitate the selection of safe and effective medications and doses. The use of PGx has been shown to be cost-effective and to decrease resource utilization in a variety of clinical settings [12,13,14]. The use of PGx data has the potential to improve engagement and communication with patients, overcome patient hesitancy related to medication trials and side effects, and improve adherence [15,16]. Patient-facing PGx material may therefore contribute to improved patient care. Some specialties, like psychiatry, may particularly benefit from PGx guidance given that psychotropic medications often take weeks to achieve efficacy, and may need to be changed in response to patients’ changing symptoms, intolerability, or inefficacy. Nevertheless, patients’ familiarity of PGx remains low, demonstrating the need for improved patient-facing PGx materials in this setting [5]. Some companies, like 23andMe, have already conducted patient comprehension studies focused on translating PGx results for individual genes. In one study, participants scored, on average, greater than 90% comprehension, and their direct-to-consumer PGx product was approved for use by the FDA based on this criterion [17].

While PGx is an emerging discipline and is becoming a standard of care, many providers have misunderstandings about its utility. This is even more of a problem for patients, who may perceive that there is a single drug that is “right” for them [18]. Pharmacological treatment is an iterative process, often requiring repeated dose adjustments or medication changes. The authors of this study, affiliated with Genomind, adapted a commercial PGx test (Genomind Professional PGx) into a report designed to be more comprehensible for consumers. The language of the PGx report was simplified to ensure that test results and clinical implications can be understood across a range of different educational levels. The original provider-facing report was consistently described as difficult to understand during clinical consultations. This study was designed to assess the readability of a new patient-facing report. We did not assess differences in readability between these two reports because they are designed for two different populations. Our primary aim was to evaluate the user comprehension of the newly developed patient-facing report, and our secondary aim was to measure changes in PGx literacy after reading through these newly developed materials using a validated PGx literacy survey.

## 2. Materials and Methods

### 2.1. Report Translation

The patient-facing report was modified from a commercially available PGx testing platform that evaluates 70 gene variants across 26 genes. This task was undertaken using a systematic approach as per the Agency for Healthcare Research and Quality (AHRQ) Health Literacy Universal Precautions Toolkit, specifically Tool 11 (“Assess, Select, and Create Easy-to-Understand Materials”) and Tool 12 (“Use Health Education Materials Effectively”) [19]. One example, consistent with the AHRQ guideline for reducing complexity through simplifying vocabulary, might take the statement “CYP2C19 poor metabolism can result in increased drug exposure, bioavailability and risk for adverse events, like QT prolongation, when taking >20 mg of citalopram,” and translate this to “Changes in your CYP2C19 gene will slow the breakdown of the antidepressant citalopram, which can lead to higher-than-expected blood levels of this drug and an increased risk for symptoms like heart palpitations,, dizziness and sudden fainting.” The initial translation of the PGx report into lay terminology was conducted by seven PharmDs and PhDs, who collectively have provided over 20,000 pharmacogenetic consults to providers and patients to date [20]. These translations were then analyzed by several readability scoring systems (e.g., Average Reading Level Consensus Calculations, Automated Readability Index, Flesch Reading Ease, Gunning Fog Index). Final readability scores for the completed patient-facing report were scored at an 8th-grade reading level using the Automated Readability Index (ARI) [21].

### 2.2. Comprehension Assessment

**Figure 1:**
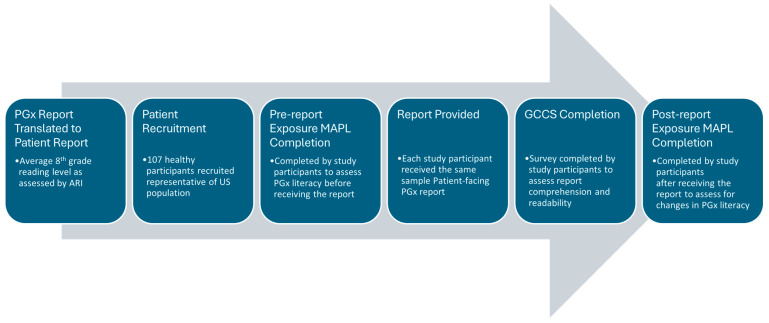
Sequence of experimental events. Abbreviations: pharmacogenetics (PGx); Minnesota Assessment of Pharmacogenomic Literacy (MAPL); Genomind consumer comprehension survey (GCCS).

A user comprehension study was then conducted from 23 February to 15 March 2024, to assess the understanding of each gene–drug interaction result in the patient-facing PGx report, which included a demographically diverse set of healthy participants. All participants lacked biomedical training and were naïve to any prior PGx testing. We developed a protocol with a market research company InsightDynamo (IDS, St. Louis Park, MN, USA), for conducting consumer comprehension testing and, prior to data collection, two rounds of pretesting were conducted to improve the comprehensibility of the protocol and survey materials, as needed. InsightDynamo recruited healthy study participants and conducted interviews structured to combine an experimental design with qualitative insight. Healthy study participants were used, since the primary aim of the study was to assess the comprehension of the patient-facing report in the average person without a medical or scientific background rather than to compare comprehension scores between different PGx reports, assess efficacy, or other clinical outcomes. The experimental design allowed for a direct quantitative comparison of outcomes related to participants’ accurate assessment of the test materials. The qualitative component explored why participants made errors when using the materials and identified report changes that could increase comprehension. One-hour group interviews with the participants were conducted, and data was collected from two sets of surveys. The primary outcome measure of report comprehension and readability was assessed by utilizing a custom report-specific survey generated by Genomind and IDS, which is referred to as the Genomind consumer comprehension survey (GCCS). The secondary outcome measure utilized the Minnesota Assessment of Pharmacogenomic Literacy (MAPL™) to compare PGx literacy before and after viewing the patient-facing report. The MAPL is considered a validated tool designed to determine the global understandability and actionability of PGx testing. Two questions concerning data privacy were removed from the MAPL, as they were considered outside the scope of the study, and the remaining questions have been described in more detail elsewhere [22]. The GCCS can be found in Supplementary File S1. The sequence of experimental steps is illustrated in Figure 1.

The group interviews were conducted by a researcher, who briefly introduced the task at hand and presented a hypothetical scenario that established a context for reviewing the report templates. Healthy participants were asked to imagine they had submitted a DNA sample (cheek swab) for genetic testing and have received the report which explains their results. The PGx report contained only mock genetic results; at no time were real participant data used, and this was clearly explained to each participant. All participants provided informed consent and were granted an IRB D2 exemption for human research under regulation 45 CFR § 46.104(d)(2). Post-survey interviews combined open-ended questions to elicit participants’ subjective understanding of the materials (e.g., “What is this report showing you?”) with closed-ended questions (e.g., “Does this report indicate a potential negative reaction to any medications?”) that probed their comprehension of the materials.

### 2.3. Participants

A total of 107 healthy participants, with an average of 52 years of age, were recruited for the study. Participant demographics are reported in Table 1. A modest financial incentive of USD 100 per survey was utilized in participant recruitment. Participants were recruited from urban and rural zip codes across the US. To ensure adequate representation of different age groups and demographics, multiple social media platforms that engage with different demographics were used for recruitment, including Facebook, LinkedIn, Twitter, and Instagram. Participants were recruited to support a mix of sex, race/ethnicity, incomes, and education levels.

*Inclusion criteria* included healthy participants between the age of 18 and 85 years with at least a secondary education, who were naïve to PGx testing.

*Exclusion criteria* included experience or expertise in genetics, healthcare, pharmaceuticals, or market research, as well as any mental health diagnosis or diagnosis of cognitive impairment that may impede comprehension of the test results (e.g., dementia, substance-induced cognitive impairment, traumatic brain injury). Exclusion criteria did not include a restriction on medication use.

### 2.4. Data Analysis

The primary analysis consisted of evaluating whether participant comprehension of the patient-facing report met the criterion of achieving a 90% or greater score (i.e., a score of 10 or greater on the 11-question GCCS), which would be consistent with a prior pharmacogenetic comprehension study used as a metric for FDA clearance [17]. Exploratory analyses were conducted to compare comprehension between various demographic groups, which utilized a z-test of proportions to analyze differences in the pre- and post-test MAPL scores, demographics, and patient comprehension scores. An original analysis was conducted using Qualtrics (XM platform) software and confirmed with a second round of analytics using SurveyStar.

The results were tabulated to provide an average comprehension score for each question in the comprehension survey. User comprehension scores > 90% are considered sufficient as per prior published methods [17]. Responses to open-ended questions were used to identify likely causes of misinterpretation.

## 3. Results

### 3.1. Survey Results and MAPL PGx Literacy

**Figure 2:**
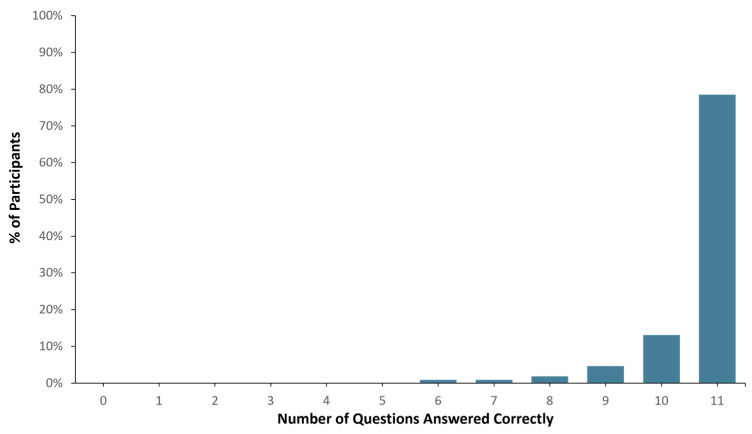
Patient report comprehension as assessed by Genomind consumer comprehension survey (GCCS).

**Figure 3:**
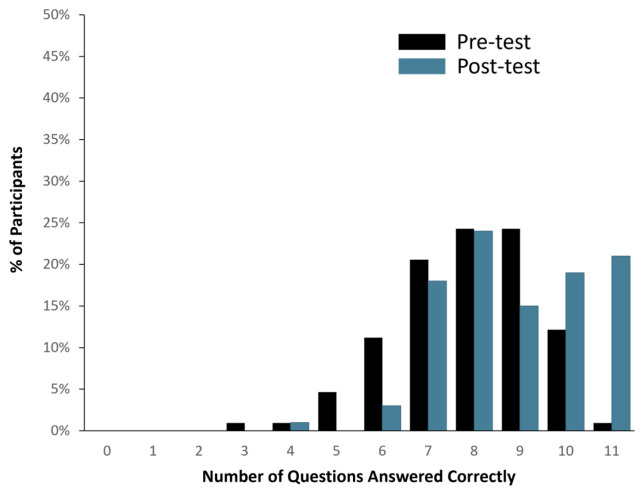
Changes in PGx literacy pre- (black bar) and post-evaluation (blue bar) of the patient-facing report, as assessed by modified MAPL.

Ninety-eight (98) out of one hundred and seven healthy study participants answered one or zero questions incorrectly, translating to a >90% comprehension score (See Figure 2). When the total number of correct answers was evaluated across all participants, 1136 out of 1177 questions were answered correctly (i.e., 96.5% accuracy). In addition, pre- and post-MAPL scores improved significantly (*p* < 0.001). More specifically, 13% of participants scored 90% or higher comprehension on the MAPL before engaging with the patient-facing report. After going through the report materials, 40% of participants scored 90% or higher in comprehension on the MAPL (see Figure 3). This significant improvement in individual question scores was observed in multiple domains including “underlying concepts” (*p* < 0.001), “limitations” (*p* = 0.002; *p* < 0.001), and “benefits” (*p* < 0.001; *p* = 0.008). There were no significant differences detected between participants when stratified by age (*p* = 0.11), sex (*p* = 0.51), race/ethnicity (*p* = 0.82), or education level (*p* = 0.16).

The questions most likely to be answered incorrectly on both the GCCS and the MAPL were found in the test limitations section/domain. More specifically, when healthy study participants were asked “Will this test, by itself, tell you if a drug matches your condition or diagnosis,” 9% of participants answered incorrectly in the GCCS. On the pre-test MAPL, 71% of participants incorrectly answered “True” when given the statement “PGx testing will tell you the best medication to treat your condition” and 54% of participants incorrectly answered “True” when given the statement “PGx testing will help determine your diagnosis.” There was a significant decrease in the percentage of incorrect answers on the MAPL after evaluating the PGx material on the patient-facing report (50.9%; *p* < 0.001 and 32%; *p* < 0.001, respectively).

### 3.2. PGx Report Modifications

The patient-facing PGx report underwent one minor modification after administering the survey to the first round of healthy study participants (n = 43). Based on participant feedback, specifically regarding the limitations of PGx testing and reasons for incorrect responses, page 1 was modified to more clearly distinguish test purpose from test limitations by including a new cautionary icon and color scheme to clarify what the testing may and may not be able to tell the patient. This modification, intended to clarify test limitations, along with representative samples of each section of the patient-facing PGx report and survey, can be found in Supplementary Figures S1 and S2, and File S1, respectively. Participants’ overall comprehension demonstrated a numerical but not statistically significant (*p* = 0.37) improvement when comparing the participants viewing report 1 versus modified report 1.1 (89% comprehension vs. 93% comprehension; Supplementary Figure S3).

## 4. Discussion

This study, which designed and evaluated a patient-facing PGx report, observed a >90% comprehension rate when assessed with healthy study participants without a biomedical background. Our results are similar to the study conducted by 23andMe, which evaluated consumer comprehension of their FDA-approved direct-to-consumer PGx report [17]. While the report itself is comprehensible to healthy participants, the data also suggest that PGx literacy is still quite low in general. More specifically, only 40% of participants scored a 90% or higher comprehension on the MAPL tool after reading through the patient-facing report, and only 13% before reading these materials. These data on PGx literacy were similar to other studies utilizing the MAPL in both patients and members of the general public, particularly in the ”test limitations” section [22,23]. This indicates that continued effort should be made to educate individuals, particularly regarding the limitations of PGx testing. Overall, this newly designed report may provide both patients and their providers with a commercial PGx report that is comprehensible to those without a biomedical background, while also increasing their overall PGx literacy.

The ability to modify and maintain patient-facing reports based on user feedback and reactions should be a key consideration during the report development process. Additionally, effective visual communication is essential for improving health literacy among patients [24]. In this study, visual displays (see Supplementary Figure S1) were added to the PGx "test limitations” section. These visual aids simplified the information and clarified the intended message, highlighting how visuals can improve patient understanding. Therefore, incorporating mechanisms for patient feedback is essential for the ongoing refinement and improvement of health-related reports, ensuring they remain clear, accurate, and effective over time.

Some providers may have reservations about patients having access to a patient-facing report and acting on the results without discussing any medication changes with their provider first. However, responses to survey questions suggest that participants understood not to make any changes to their medications. Consistent with a shift toward patient-centered care, this can promote an active collaboration between the patient and their provider. Patient engagement in their healthcare is a key driver in cost reduction, securing an effective use of resources, and ensuring patient–provider satisfaction [25]. Future studies should be conducted to ensure that patients who receive these results can understand their report, as well as to determine how likely they are to follow instructions for discussing these results with their prescriber prior to changing their current medication regimen. By understanding the nature of genetic results and providing direct links to published PGx guidelines, patients should be able to bring their results to any prescriber, not just the prescriber that originally ordered the test, allowing for the broader applicability of these results across different specialties.

The limitations of the current research include the use of only healthy participants, who may not be representative of the most common population tested with PGx. Multi-panel PGx tests require an order by a prescriber, and most 3rd-party insurers require a diagnosis for some type of health condition for coverage. Several common diagnoses, such as major depressive disorder or generalized anxiety disorder, are sometimes associated with cognitive impairments and may impact report interpretation [26,27]. Many of the participants identified as white and female, which may limit the generalizability of the data. However, participants’ race/ethnicity in this study population was similar to the frequency found in the general US population. While using social media platforms as a recruitment tool could have introduced selection bias, study authors tried to account for this by including participants of lower economic status/background, who may have more limited access to these platforms. Our secondary analysis investigating differences between patient demographic characteristics like sex, education level, and ethnicity may be insufficiently powered to detect a difference, given the smaller sample size between groups. Future studies should examine subject comprehension using larger sample sizes in underrepresented populations.

In summary, we found that translating PGx test results into lay language may provide individuals with a greater understanding of how their DNA may impact medications. This understanding could facilitate discussions between patients and providers, potentially leading to improved patient care by increasing engagement in treatment [3,16]. Lastly, there is a great need to provide ongoing education to individuals on what information PGx testing can and cannot provide. Given that >70% of the participants in this study believed PGx testing can choose the perfect medication, and >50% of participants believed the test would help determine their diagnosis, these data suggest that the general population may have a poor understanding of pharmacogenetics. Gathering patient feedback and redesigning reports to be more patient friendly has great potential to mitigate this knowledge gap.

## Figures and Tables

**Table 1:** Participant demographics.

Demographic Category	Frequency (%)
**Age**	
25–34	20 (19)
35–44	14 (13)
45–54	22 (21)
55–64	22 (21)
65+	29 (27)
**Gender**	
Male	36 (34)
Female	71 (66)
**Race**	
White	72 (68)
Black	13 (12)
Asian	9 (8)
Native American	2 (2)
Other	11 (10)
**Ethnicity**	
Hispanic/Latino	25 (23)
**Education Level**	
High School	16 (15)
Some College	30 (28)
Associate Degree in College (2-Year)	7 (7)
Bachelor’s Degree in College (4-Year)	39 (36)
Graduate or Professional Degree	15 (14)

## Data Availability

The raw data supporting the conclusions of this article will be made available by the authors on request.

## Supplementary Materials

The following supporting information can be downloaded at: https://www.mdpi.com/article/10.3390/jpm15060247/s1, Figure S1: Patient-facing report updates made to clarify test limitations, Figure S2: Representative sections of the Patient-facing report, File S1: Genomind consumer comprehension survey (GCCS), Figure S3: Overall comprehension by report modification.
