# Supplementary material for: Assessing the Impact of Simplified Language on a Patient-Facing Pharmacogenetic Report: A User Comprehension Study
Source: J Pers Med. 2025 Jun 12;15(6):247. doi: 10.3390/jpm15060247 (PMC12194072; doi:10.3390/jpm15060247)

Supplementary Figure S1.

Patient-facing report updates made to clarify test limitations

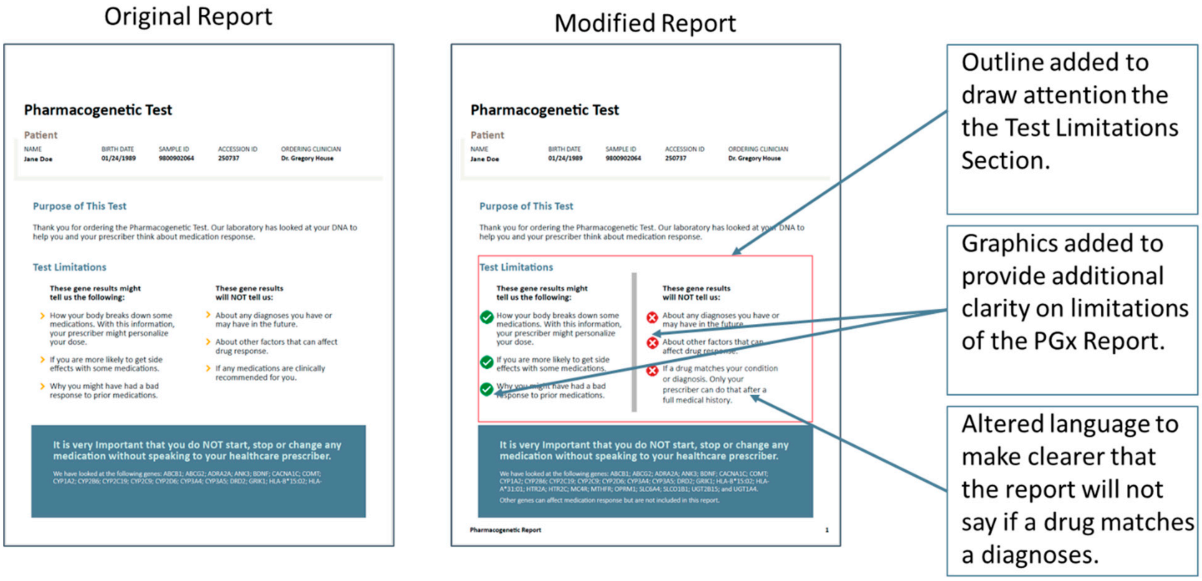

Supplementary Figure S2.

Representative sections of the Patient-facing report

# Pharmacogenetic Test

## Patient

| NAME | BIRTH DATE | SAMPLE ID | ACCESSION ID | ORDERING CLINICIAN |
|---|---|---|---|---|
| **Jane Doe** | **01/24/1989** | **9800902064** | **250737** | **Dr. Gregory House** |

## Purpose of This Test

Thank you for ordering the Pharmacogenetic Test. Our laboratory has looked at your DNA to help you and your prescriber think about medication response.

## Test Limitations

**These gene results might tell us the following:**

- How your body breaks down some medications. With this information, your prescriber might personalize your dose.
- If you are more likely to get side effects with some medications.
- Why you might have had a bad response to prior medications.

**These gene results will NOT tell us:**

- About any diagnoses you have or may have in the future.
- About other factors that can affect drug response.
- If a drug matches your condition or diagnosis. Only your prescriber can do that after a full medical history.

**It is very Important that you do NOT start, stop or change any medication without speaking to your healthcare prescriber.**

We have looked at the following genes: ABCB1; ABCG2; ADRA2A; ANK3; BDNF; CACNA1C; COMT; CYP1A2; CYP2B6; CYP2C19; CYP2C9; CYP2D6; CYP3A4; CYP3A5; DRD2; GRIK1; HLA-B*15:02; HLA-A*31:01; HTR2A; HTR2C; MC4R; MTHFR; OPRM1; SLC6A4; SLCO1B1; UGT2B15; and UGT1A4.

Other genes can affect medication response but are not included in this report.

**Pharmacogenetic Report** 1

## How to Read Your Report

**SECTION 1 is organized by gene.**
**It is divided into 3 parts:**

**1A. Genes with Important Effect:** Gene types that you should discuss with your healthcare prescriber. These genes can affect some drugs and have specific guidelines. If you are taking any of the drugs listed, you should let your prescriber know.

**1B. Genes with Moderate Effect:** Gene types that are informative for certain drugs. These might help explain prior drug response or guide new treatments.

**1C. Genes with Normal Effect:** Gene types that are normal. They have no known effect on any drugs.

**SECTION 2 is organized by medications.**

**2. Medications Affected by Your Genes:** Medications that should be discussed with your prescriber because of your gene type. Medications are sorted by drug class (for example: heart medications, pain medications)

## Disclaimer

**It is very Important that you do NOT start, stop or change any medication without speaking to your healthcare prescriber.**

This report is made to help you and your prescriber make decisions regarding the use, dosing and safety of drugs based on your DNA. However, this is not the only information needed to make decisions about your medications.

You and your prescriber must consider these gene results along with all of your other medical background.

Our laboratory results have been shown to have an accuracy rate up to 99.9%, but it is still possible that results can be wrong.

For information about our test methodology, click here.

It is possible that these gene results reveal non-paternity.

**Many other factors can affect drug response, including but not limited to age, weight, health status and history, medications, and food.**

SECTION 1A

## Genes with Important Effect

Section 1A shows your gene results that **may have an important effect on some drugs** and are **associated with specific drug recommendations** with strong evidence.

| Gene | Your Version of the Gene | How this Could Affect Medications |
|---|---|---|
| **CYP2C9**<br>This gene controls the break-down of some drugs. | **CYP2C9 IM (Intermediate Metabolizer)**<br>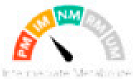<br>See How Common Your Gene Type Is | This CYP2C9 IM gene type can lead to more drug in your body. There is specific guidance for the following medications:<br><br>Cerebyx (fosphenytoin)<br>Coumadin (warfarin)<br>Dilantin (phenytoin)<br>Lescol (fluvastatin)<br>Marinol (dronabinol)<br>Mayzent (siponimod)<br><br>**Non-steroidal anti-inflammatory drugs (NSAIDs):**<br>Motrin (ibuprofen)<br>Celebrex (celecoxib)<br>Feldene (piroxicam)<br>Mobic (meloxicam)<br>Ansaid (flurbiprofen)<br><br>If you are taking or considering these medications, let your prescriber know about these gene results.<br><br>Click here for a list of other drugs that may also be affected by this gene. |
| **CYP2C19**<br>This gene controls the break-down of some drugs. | **CYP2C19 PM (Poor Metabolizer)**<br>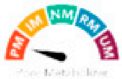<br>See How Common Your Gene Type Is | This CYP2C19 PM gene type can change the amount of drug in your body. There is specific guidance for the following medications:<br><br>Addyi (flibanserin)<br>Briviact (brivaracetam)<br>Onfi (clobazam)<br>Plavix (clopidogrel)<br>Soma (carisoprodol)<br>Valium (diazepam)<br>Vfend (voriconazole)<br><br>**Selective serotonin reuptake inhibitors (SSRIs):**<br>Celexa (citalopram)<br>Lexapro (escitalopram)<br>Zoloft (sertraline)<br><br>**Proton pump inhibitors (PPIs):**<br>Dexilant (dexlansoprazole)<br>Prevacid (lansoprazole)<br>Protonix (pantoprazole)<br>Prilosec (omeprazole)<br><br>**Tricyclic antidepressants (TCAs):**<br>Anafranil (clomipramine)<br>Elavil (amitriptyline)<br>Silenor (doxepin)<br>Tofranil (imipramine)<br>Surmontil (trimipramine)<br><br>If you are taking or considering these medications, let your prescriber know about these gene results.<br><br>Click here for a list of other drugs that may also be affected by this gene. |
| **HLA-A 31:01**<br>This gene is linked to serious skin rashes with some drugs used for seizures or bipolar disorder. | **HLA-A 31:01 Positive**<br>See How Common Your Gene Type Is | People with this HLA-A 31:01 Positive gene type have an increased risk for a serious rash with the following medication:<br><br>Tegretol (carbamazepine)<br><br>**It could be dangerous for you to take this medication.** If you are taking this medicine and have been taking it for less than 3 months, you may need to consider stopping it. Talk to your prescriber before changing or stopping your medication.<br><br>Contact your prescriber immediately if you are taking this medicine and you develop a rash. |

| Medication | How Your Genes Can Affect this Medication | Guidance for Your Prescriber |
|---|---|---|
| **Medications for Pain** | | |
| **Mobic (meloxicam)** | Your CYP2C9 IM gene type can increase the amount of this drug in your body. This could increase your risk of side effects.<br>Speak with your prescriber about gene guided dosing or other drug options or if you are having side effects with this medication. | |
| **Feldene (piroxicam)** | Your CYP2C9 IM gene type can increase the amount of this drug in your body. This could increase the risk of side effects.<br>Speak with your prescriber about other options or if you are having side effects with this medication. | |
| **Soma (carisoprodol)** | Your CYP2C19 IM gene type can increase the amount of this drug in your body.<br>Speak with your prescriber if you are having side effects with this medication. | |
| **Medications for Cholesterol** | | |
| **Lescol (fluvastatin)** | Your SLCO1B1 NF and CYP2C9 IM gene type can increase the amount of this drug in your body. This could increase your risk of side effects, such as muscle weakness.<br>If you are taking 40 mg or more and having side effects, speak with your prescriber about gene guided dosing. | |
| **Medications for Your Stomach/Digestion** | | |
| **Proton pump inhibitors (PPIs): Dexilant (dexlansoprazole), Prevacid (lansoprazole), Prilosec (omeprazole), Protonix (pantoprazole)** | Your CYP2C19 PM gene type can increase the amount of these drugs in your body. This could increase the risk of side effects.<br>Speak with your prescriber about gene guided dosing if you need treatment for longer than 12 weeks or if you are having side effects to these medications. | |
| **Medications for Seizure Disorders** | | |
| **Cerebyx (fosphenytoin), Dilantin (phenytoin)** | Your CYP2C9 IM gene type can increase the amount of these drugs in your body. This could increase the risk of side effects.<br>Speak with your prescriber about gene guided dosing or if you are having side effects with these medications. | |
| **Onfi (clobazam)** | Your CYP2C19 IM gene type can increase the amount of this drug in your body. This could increase the risk of side effects.<br>If you are taking 20 mg or more, speak with your prescriber about gene guided dosing. | |
| **Briviact (brivaracetam)** | Your CYP2C19 IM gene type can increase the amount of this drug in your body. This could increase the risk of side effects.<br>Speak with your prescriber if you are having side effects with this medication. | |

Supplementary File S1.

Genomind consumer comprehension survey (GCCS)

Please use the included report titled **Pharmacogenetic Test** to answer following questions:

This is a generic report and does not contain information about you. It is an example report that a person might receive after sending a DNA sample in for Pharmacogenetic testing.

1. Looking at **Page 1** of this test report, what is the purpose of this genetic test?
    - To explore your DNA to help you and your doctor think about medication response.
    - To explore your DNA to better understand your ethnicity.
    - To explore your DNA to find out if you have any diseases.
    - To explore your DNA and recommend an appropriate diet.

2. **Page 1** includes a section called **Test Limitations**. Looking at that section, please answer the following questions:
    a. Can this test tell you if you are at risk for any health conditions?
        - Yes
        - No
    b. Can this test tell you if any medications are clinically recommended for you?
        - Yes
        - No
    c. Can this test tell you how you personally break down some medications?
        - Yes
        - No
    d. Can this test tell you if you are more likely to have side effects to some medications?
        - Yes
        - No

3. Looking at **Page 1**, what action is recommended for you to take with your medications?
    - It is important that you stop taking all medications.
    - It is very important that you stop taking some medications.
    - It is important that you begin taking new medications.
    - It is very important that you do NOT start, stop, or change any medication without speaking to your healthcare prescriber.

4. **Page 3** has a section called **Disclaimer.** Looking at that section, can your age and weight affect medication response?
    - Yes
    - No

5. On **Page 4**, the gene **HLA-A 31:01** is positive. As a result, there is an increased risk for a serious rash with what medication?
    - Aspirin (acetaminophen)
    - Tegretol (carbamazepine)
    - Coumadin (warfarin)
    - Lescol (fluvastatin)

6. On **Page 7**, what does this report say about your potential risk with the drug **Plavix (clopidogrel)**?
    - There is no potential risk with this drug
    - The CYP2C19 PM gene can reduce the effectiveness of this drug and could lead to an increase in heart attacks or stroke.
    - This drug could cause a side effect such as muscle weakness.
    - This drug may cause upset stomach and diarrhea.

7. On **Page 4**, Gene CYP2C9 is labeled with this graphic:

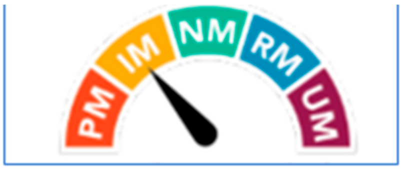


What does the graphic tell you?

- Gene CYP2C9 an **Ultra-Rapid Metabolizer** which can lead to less drug in your body.
- Gene CYP2C9 is an **Intermediate Metabolizer** which can lead to more drug in your body.

- o Gene CYP2C9 is a **Normal Metabolizer** which leads to a normal amount of drug in your body.
- o Gene CYP2C9 has an **unknown effect** on your ability to metabolize drugs.

8. On what page is there a section labeled “**Ready to Learn More**?” where you can learn more about Pharmacogenetics?
   - o Page 1
   - o Page 3
   - o Page 6
   - o Page 9.

**Qualitative Questions:**

Overall, how easy or difficult was it to find the answers to these questions in your example report?

Was anything confusing about the report or about this test?

Supplemental Figure S3

Overall comprehension by report modification

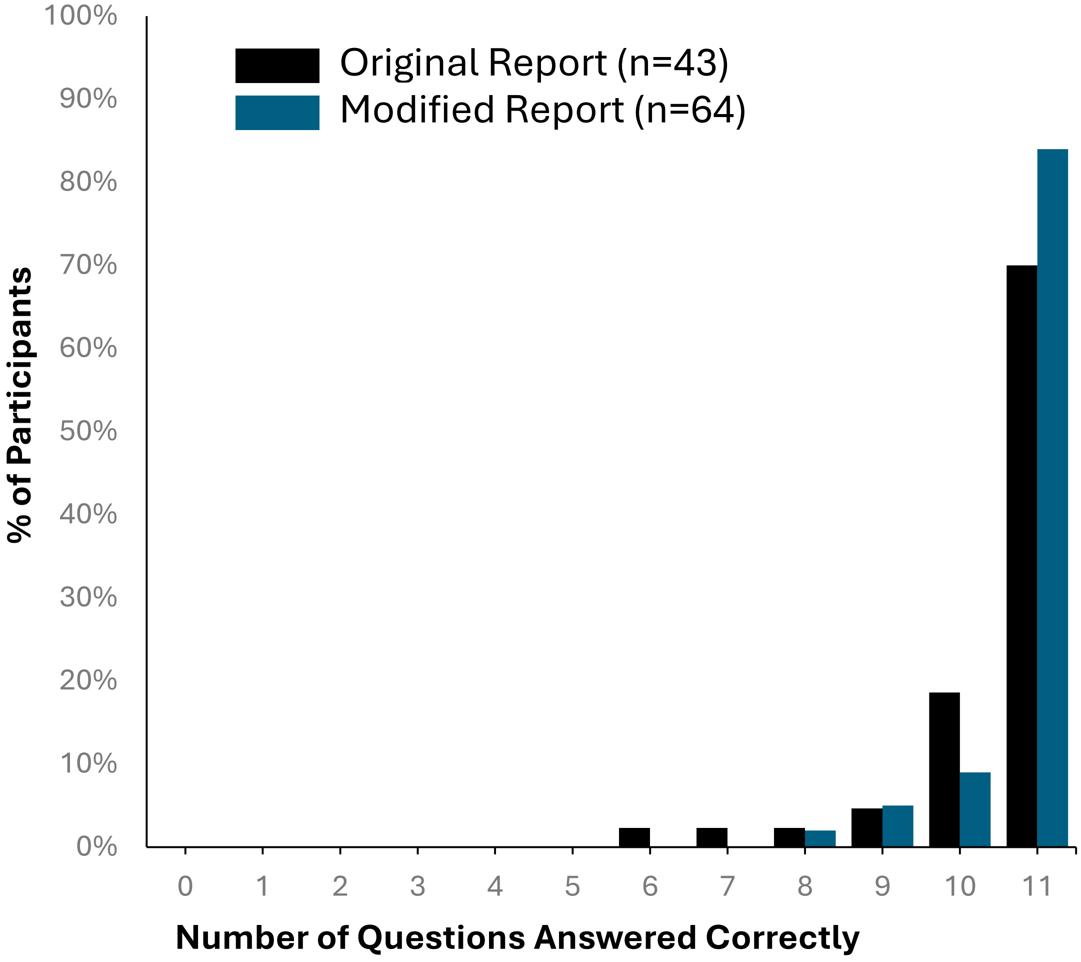

Supplement: Supplementary file 1 [file jpm-15-00247-s001.zip › jpm-3635468-supplementary.pdf]
